# Supplementary material for: Projections of epidemic transmission and estimation of vaccination impact during an ongoing Ebola virus disease outbreak in Northeastern Democratic Republic of Congo, as of Feb. 25, 2019
Source: PLoS Negl Trop Dis. 2019 Aug 5;13(8):e0007512. doi: 10.1371/journal.pntd.0007512 (PMC6695208; doi:10.1371/journal.pntd.0007512)
Supplement: S1 Table — Official reported case counts for each epidemic are given, including suspected cases (“Reported Count”). Case counts for the time series data included in the models include only probable and confirmed cases (“Time Series Count”). Case counts for historic outbreaks were pulled from publicly available literature [17–30]. Lastly, each historic outbreak’s inclusion in the regression, stochastic, and auto-regression models is enumerated. (PDF) [file pntd.0007512.s001.pdf]

Time Period	Country	Reported Count	Time Series Count	Regression?	Stochastic?	Auto-Regression?
Aug–Sep 1976	DRC*	318	262	Yes	No	Yes
Jun–Nov 1976	Sudan	284	284	Yes	No	Yes
Aug–Sep 1979	Sudan	34	34	Yes	Yes	Yes
Dec 1994–Feb 1995	Gabon	52	49	Yes	No	Yes
May–Jul 1995	DRC	315	317	Yes	Yes	Yes
Jan–Apr 1996	Gabon	37	29	Yes	Yes	Yes
Jul 1996–Mar 1997	Gabon	60	–	No	No	No
Oct 2000–Jan 2001	Uganda	425	436	Yes	No	Yes
Oct 2001–Jul 2002	Gabon, Republic of the Congo	124	124	Yes	Yes	Yes
Dec 2002–Mar 2003	Republic of the Congo	143	–	No	No	No
Nov–Dec 2003	Republic of the Congo	35	35	Yes	Yes	Yes
Apr–Jun 2004	Sudan	17	17	Yes	Yes	Yes
Apr–May 2005	DRC	12	12	Yes	Yes	Yes
Aug–Nov 2007	DRC	264	264	Yes	Yes	Yes
Dec 2007–Jan 2008	Uganda	131	127	Yes	Yes	Yes
Dec 2008–Feb 2009	DRC	32	32	Yes	Yes	Yes
Jun–Aug 2012	Uganda	24	24	Yes	Yes	Yes
Jun–Nov 2012	DRC	52	52	Yes	Yes	Yes
Aug–Nov 2014	DRC	66	62	Yes	Yes	Yes
Jul–Oct 2014	Nigeria (offshoot of West African outbreak)	20	–	No	No	No
Jan 2014–Jun 2016	Guinea, Liberia, Sierra Leone	28,616	21,422	Yes	No	Yes
Apr–Jun 2018	DRC	53	53	Yes	Yes	Yes

*Democratic Republic of Congo (formerly Zaïre)
