# Supplementary material for: Projections of epidemic transmission and estimation of vaccination impact during an ongoing Ebola virus disease outbreak in Northeastern Democratic Republic of Congo, as of Feb. 25, 2019
Source: PLoS Negl Trop Dis. 2019 Aug 5;13(8):e0007512. doi: 10.1371/journal.pntd.0007512 (PMC6695208; doi:10.1371/journal.pntd.0007512)
Supplement: S2 Table — This table summarizes the medians and 95% prediction intervals produced by each model on the most recent data set included, and their probabilities of the large outcomes discussed in the text. (PDF) [file pntd.0007512.s002.pdf]

**Forecast as of**	**Forecast**	**Model**	**Lower**	**Median**	**Upper**	**Size 1,000**	**Size 10,000**	**Size 28,616**
2019-02-25	2019-03-04	Auto-Regression	876	883	908	0.00002	0.00000	0.00000
2019-02-25	2019-03-04	Stochastic	868	920	1028	0.11077	0.00000	0.00000
2019-02-25	2019-03-11	Auto-Regression	876	889	933	0.00204	0.00000	0.00000
2019-02-25	2019-03-11	Stochastic	872	933	1054	0.16451	0.00000	0.00000
2019-02-25	2019-03-25	Auto-Regression	877	898	983	0.03327	0.00000	0.00000
2019-02-25	2019-03-25	Stochastic	874	955	1105	0.26913	0.00000	0.00000
2019-02-25	2019-04-22	Auto-Regression	877	915	1095	0.16126	0.00000	0.00000
2019-02-25	2019-04-22	Stochastic	874	976	1180	0.39925	0.00000	0.00000
2019-02-25	final	Gott's Rule	921	1749	17498	0.86625	0.07875	0.02183
2019-02-25	final	Stochastic	860	990	1295	0.46620	0.00000	0.00000
2019-02-25	final	Theil-Sen Regression	882	953	1136	0.29805	0.00000	0.00000
