# Supplementary material for: Projections of epidemic transmission and estimation of vaccination impact during an ongoing Ebola virus disease outbreak in Northeastern Democratic Republic of Congo, as of Feb. 25, 2019
Source: PLoS Negl Trop Dis. 2019 Aug 5;13(8):e0007512. doi: 10.1371/journal.pntd.0007512 (PMC6695208; doi:10.1371/journal.pntd.0007512)
Supplement: S1 Fig — We retained snapshots of the data set of case counts to date as of multiple dates of data collection, for use in scoring of retrospective model projections against known subsequent counts. In later data sets, due to the larger number of data points, a subset of the case counts was selected for use in the stochastic model’s particle filtering step, as noted in the figure. Where not otherwise noted, all case counts shown were used in the stochastic model’s particle filtering step. (PDF) [file pntd.0007512.s003.pdf]

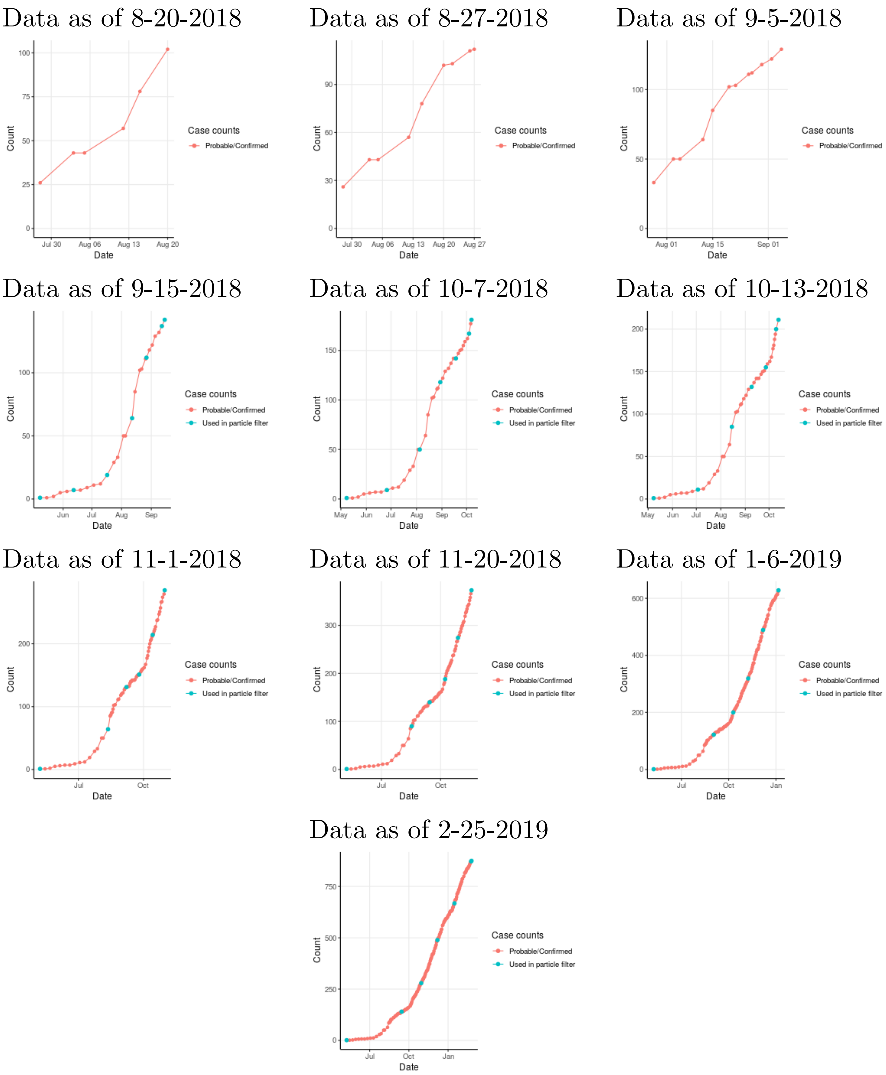
Data as of 8-20-2018
Data as of 8-27-2018
Data as of 9-5-2018
Data as of 9-15-2018
Data as of 10-7-2018
Data as of 10-13-2018
Data as of 11-1-2018
Data as of 11-20-2018
Data as of 1-6-2019
Data as of 2-25-2019
Case counts
Probable/Confirmed
Used in particle filter
Count
Date
