# Supplementary figures and images for: Projections of epidemic transmission and estimation of vaccination impact during an ongoing Ebola virus disease outbreak in Northeastern Democratic Republic of Congo, as of Feb. 25, 2019
Source: PLoS Negl Trop Dis. 2019 Aug 5;13(8):e0007512. doi: 10.1371/journal.pntd.0007512 (PMC6695208; doi:10.1371/journal.pntd.0007512)

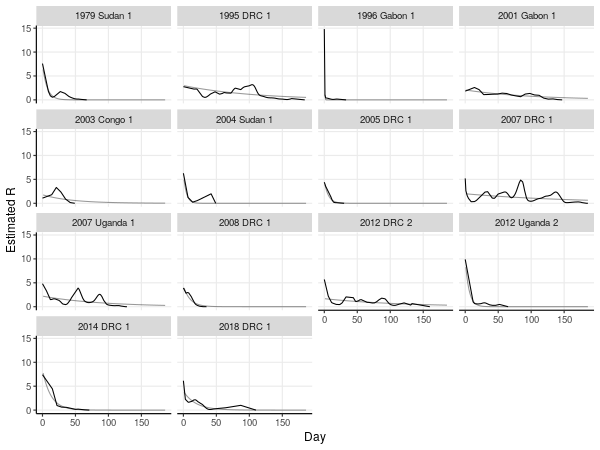

Supplement: S2 Fig — Epidemic curves reported for past Ebola outbreaks were used to estimate time series of effective reproduction number (R) by day, which were then fit to an exponentially declining (“quenched”) curve. The quenching rate parameter τ estimates the relative change in R per day from Rinitial that results from outbreak control efforts, behavioral changes in response to the outbreak, and potential local depletion of susceptibles. Estimates of R by day are drawn as heavy curves, and exponentially quenched curves R = Rinitiale−τd fit to each series of R estimates are drawn as lighter curves. (PNG) [file pntd.0007512.s004.png]

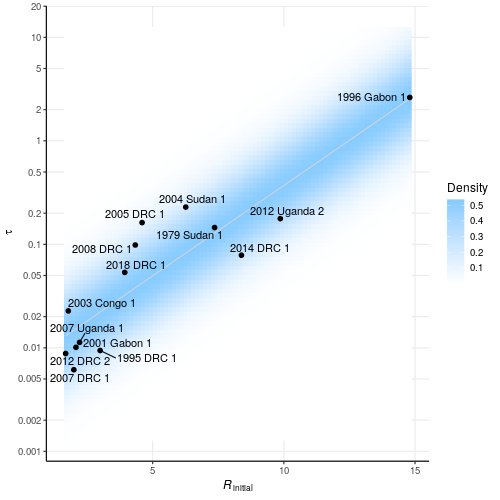

Supplement: S3 Fig — The parameters Rinitial and τ estimated by that curve fitting on past epidemics (S2 Fig) were used to create a distribution from which values were sampled to parametrize the stochastic simulation. Black dots are pairs Rinitial and quenching rate τ estimated from past Ebola outbreaks, and blue cloud is the continuous distribution from which pairs are sampled for simulation. (PNG) [file pntd.0007512.s005.png]

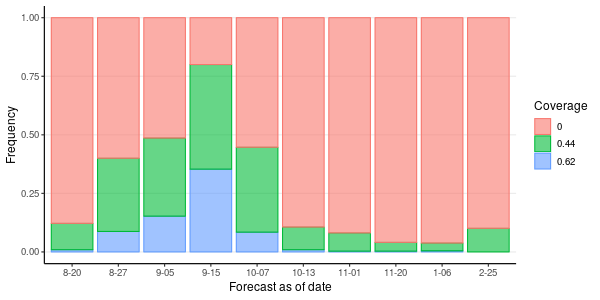

Supplement: S5 Fig — Note that each bar of this plot is an estimate based on the entire time series as of the date indicated, that is, they are cumulative estimates of the overall vaccine coverage, not successive estimates of short-term coverage. (PNG) [file pntd.0007512.s007.png]
