# Supplementary material for: Projections of epidemic transmission and estimation of vaccination impact during an ongoing Ebola virus disease outbreak in Northeastern Democratic Republic of Congo, as of Feb. 25, 2019
Source: PLoS Negl Trop Dis. 2019 Aug 5;13(8):e0007512. doi: 10.1371/journal.pntd.0007512 (PMC6695208; doi:10.1371/journal.pntd.0007512)
Supplement: S4 Fig — The Rinitial and τ parameters driving simulated outbreaks that were successful in passing the particle filtering step, which selects simulated outbreaks that match the reported case counts, tended to cluster in particular locations within the assumed distribution. In some cases, distinct ranges of Rinitial and/or τ were selected in conjunction with the different vaccine coverage scenarios. Shown here is the distribution of parameter combinations (Rinitial, τ) selected by the filtering process, colored by vaccine coverage scenario, for successive snapshots of available case count data. As in previous figure, black dots represent Rinitial, τ pairs estimated for past outbreaks (for comparison), and colors illustrate the density of Rinitial, τ pairs selected by filtering simulated outbreaks, by level of vaccine coverage. In the Feb. 25 dataset, no simulated outbreaks with vaccine coverage at the 62% (high) level were selected. (PDF) [file pntd.0007512.s006.pdf]

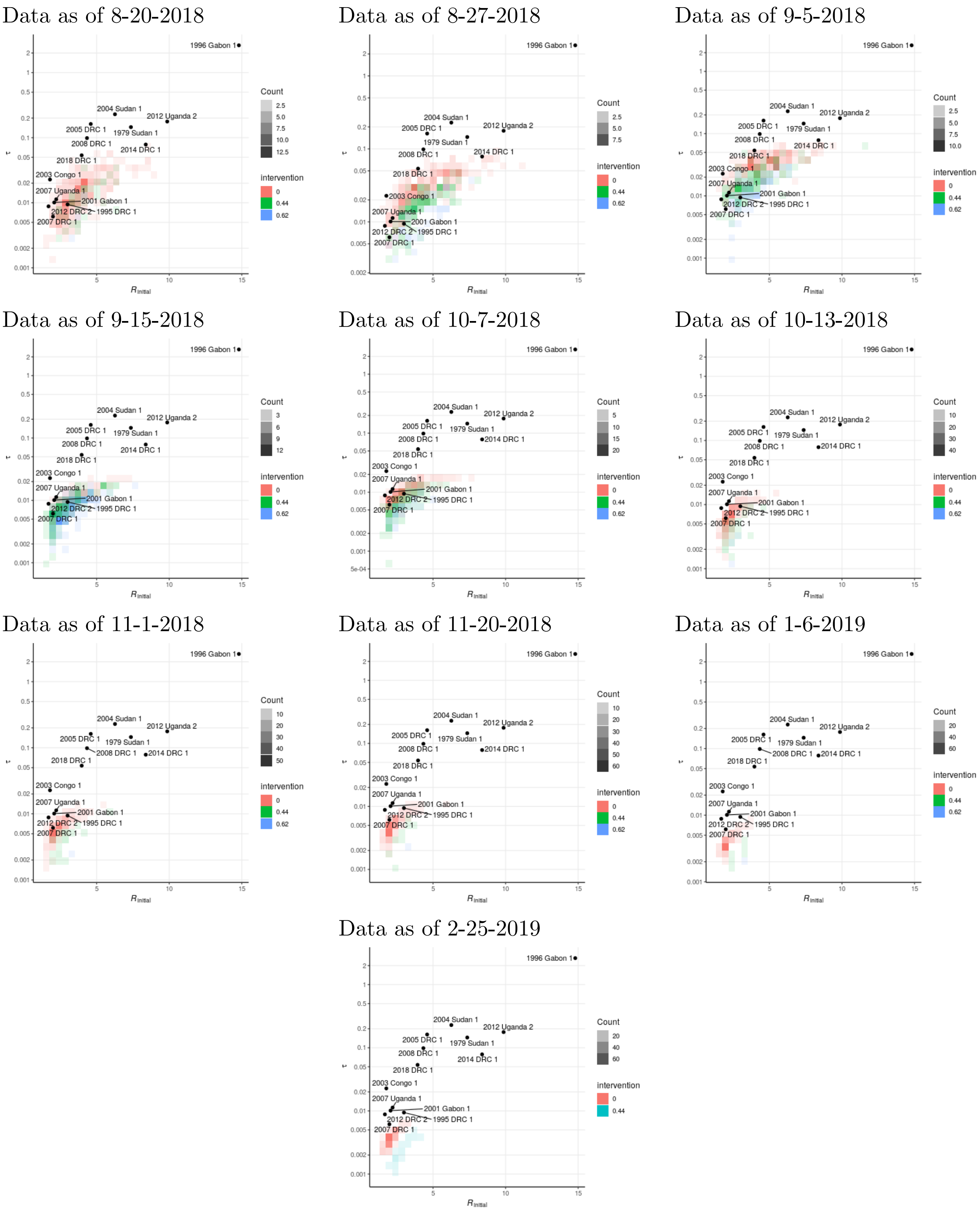

Data as of 8-20-2018
Data as of 8-27-2018
Data as of 9-5-2018
Data as of 9-15-2018
Data as of 10-7-2018
Data as of 10-13-2018
Data as of 11-1-2018
Data as of 11-20-2018
Data as of 1-6-2019
Data as of 2-25-2019
Count
intervention
0
0.44
0.62
τ
R_{initial}
1996 Gabon 1
2004 Sudan 1
2012 Uganda 2
2005 DRC 1
1979 Sudan 1
2008 DRC 1
2014 DRC 1
2018 DRC 1
2003 Congo 1
2007 Uganda 1
2001 Gabon 1
2012 DRC 2
1995 DRC 1
2007 DRC 1
