# Supplementary material for: Projections of epidemic transmission and estimation of vaccination impact during an ongoing Ebola virus disease outbreak in Northeastern Democratic Republic of Congo, as of Feb. 25, 2019
Source: PLoS Negl Trop Dis. 2019 Aug 5;13(8):e0007512. doi: 10.1371/journal.pntd.0007512 (PMC6695208; doi:10.1371/journal.pntd.0007512)

Data as of 8-20-2018

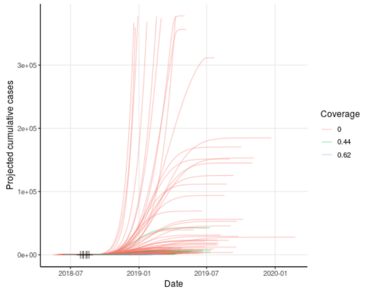

Data as of 8-27-2018

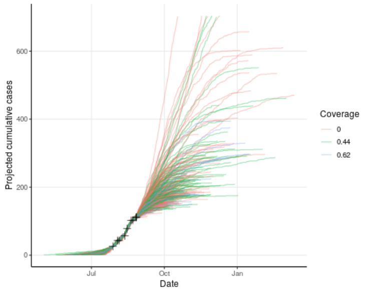

Data as of 9-5-2018

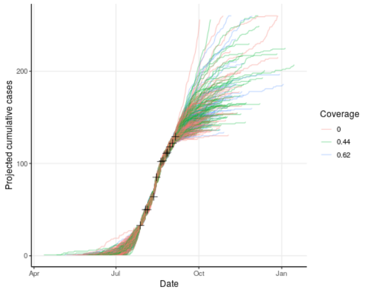

Data as of 9-15-2018

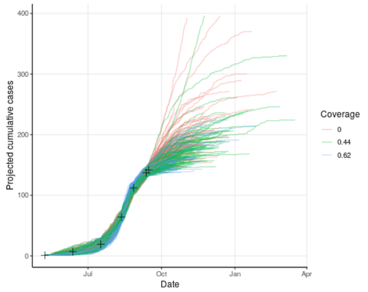

Data as of 10-7-2018

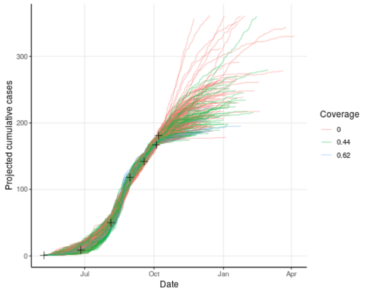

Data as of 10-13-2018

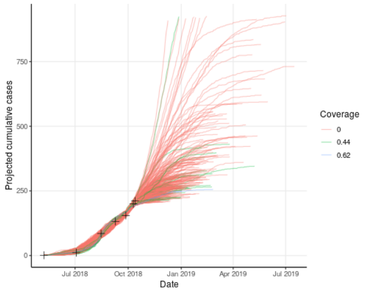

Data as of 11-1-2018

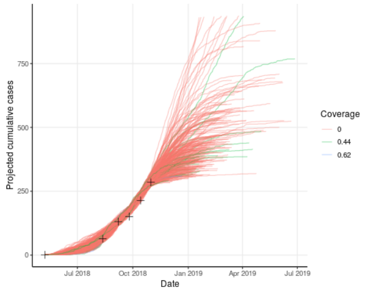

Data as of 11-20-2018

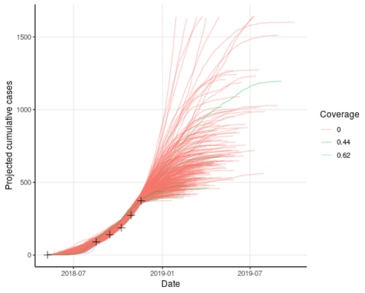

Data as of 1-6-2019

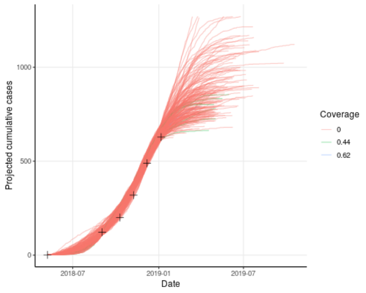

Data as of 2-25-2019

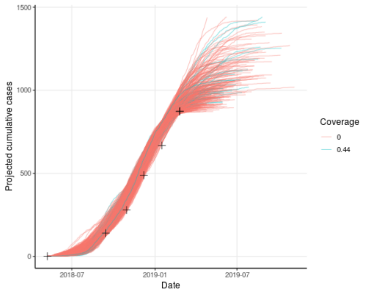

Supplement: S6 Fig — The simulations passing the particle filtering step, representing a distribution of parameter values and vaccine scenarios, were continued beyond the particle filtering points to generate a spreading set of projections of case counts at later dates, shown here. This sample of projected case counts by day was smoothed to create probabilistic projections of projected case counts at the desired future dates. The vertical axis is cut off at the upper limit of the 95% prediction interval for outbreak sizes, for readability. The 62% (high) vaccine coverage scenario is not represented in the February 25 ensemble due to the action of the filtering step of the model. (PDF) [file pntd.0007512.s008.pdf]
