# Supplementary material for: Projections of epidemic transmission and estimation of vaccination impact during an ongoing Ebola virus disease outbreak in Northeastern Democratic Republic of Congo, as of Feb. 25, 2019
Source: PLoS Negl Trop Dis. 2019 Aug 5;13(8):e0007512. doi: 10.1371/journal.pntd.0007512 (PMC6695208; doi:10.1371/journal.pntd.0007512)

Data as of 8-20-2018

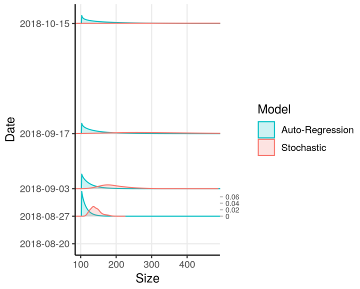

Data as of 8-27-2018

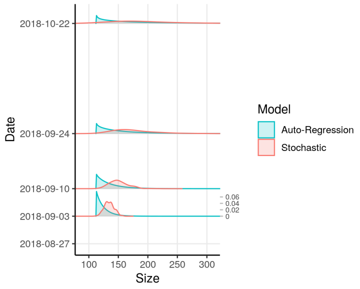

Data as of 9-5-2018

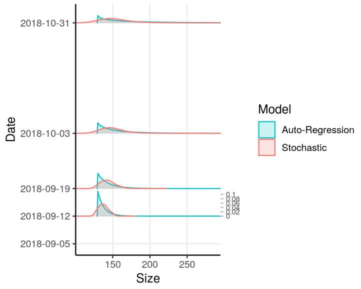

Data as of 9-15-2018

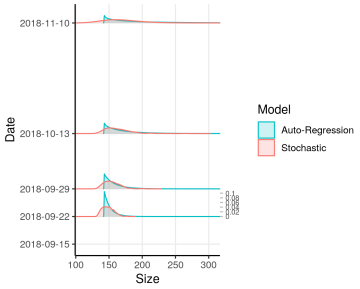

Data as of 10-7-2018

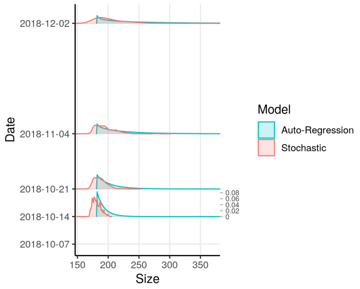

Data as of 10-13-2018

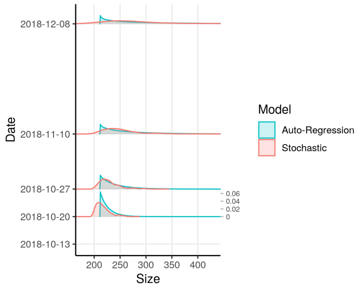

Data as of 11-1-2018

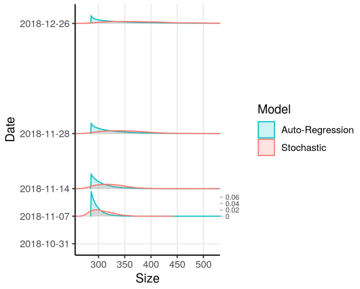

Data as of 11-20-2018

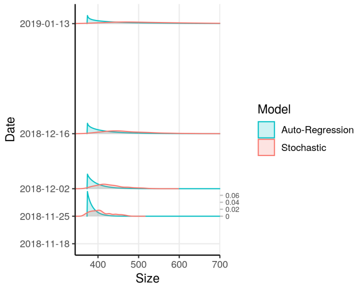

Data as of 1-6-2019

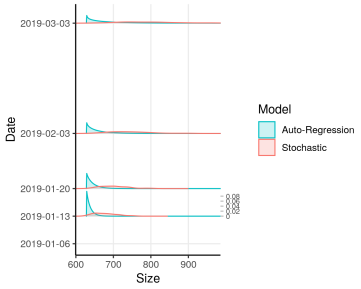

Data as of 2-25-2019

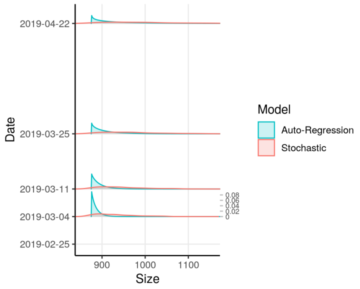

Supplement: S7 Fig — Probability scale (marked on right) is consistent across dates within each subplot. (PDF) [file pntd.0007512.s009.pdf]
