# Supplementary material for: Projections of epidemic transmission and estimation of vaccination impact during an ongoing Ebola virus disease outbreak in Northeastern Democratic Republic of Congo, as of Feb. 25, 2019
Source: PLoS Negl Trop Dis. 2019 Aug 5;13(8):e0007512. doi: 10.1371/journal.pntd.0007512 (PMC6695208; doi:10.1371/journal.pntd.0007512)
Supplement: S8 Fig — (PDF) [file pntd.0007512.s010.pdf]

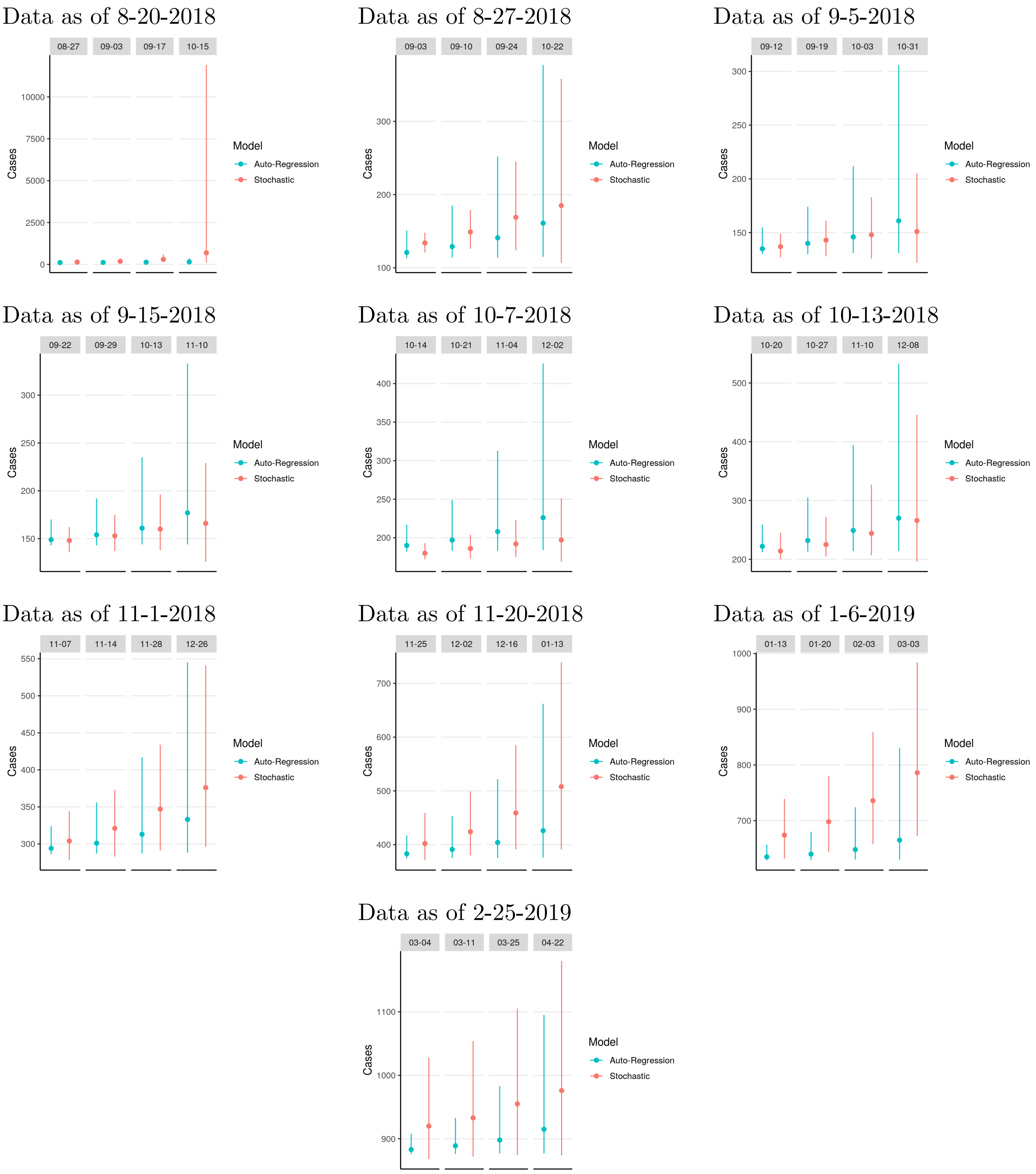
Data as of 8-20-2018
08-27
09-03
09-17
10-15
Cases
10000
7500
5000
2500
0
Model
Auto-Regression
Stochastic
Data as of 8-27-2018
09-03
09-10
09-24
10-22
Cases
300
200
100
Model
Auto-Regression
Stochastic
Data as of 9-5-2018
09-12
09-19
10-03
10-31
Cases
300
250
200
150
Model
Auto-Regression
Stochastic
Data as of 9-15-2018
09-22
09-29
10-13
11-10
Cases
300
250
200
150
Model
Auto-Regression
Stochastic
Data as of 10-7-2018
10-14
10-21
11-04
12-02
Cases
400
350
300
250
200
Model
Auto-Regression
Stochastic
Data as of 10-13-2018
10-20
10-27
11-10
12-08
Cases
500
400
300
200
Model
Auto-Regression
Stochastic
Data as of 11-1-2018
11-07
11-14
11-28
12-26
Cases
550
500
450
400
350
300
Model
Auto-Regression
Stochastic
Data as of 11-20-2018
11-25
12-02
12-16
01-13
Cases
700
600
500
400
Model
Auto-Regression
Stochastic
Data as of 1-6-2019
01-13
01-20
02-03
03-03
Cases
1000
900
800
700
Model
Auto-Regression
Stochastic
Data as of 2-25-2019
03-04
03-11
03-25
04-22
Cases
1100
1000
900
Model
Auto-Regression
Stochastic
