# Supplementary material for: Projections of epidemic transmission and estimation of vaccination impact during an ongoing Ebola virus disease outbreak in Northeastern Democratic Republic of Congo, as of Feb. 25, 2019
Source: PLoS Negl Trop Dis. 2019 Aug 5;13(8):e0007512. doi: 10.1371/journal.pntd.0007512 (PMC6695208; doi:10.1371/journal.pntd.0007512)
Supplement: S9 Fig — (PDF) [file pntd.0007512.s011.pdf]

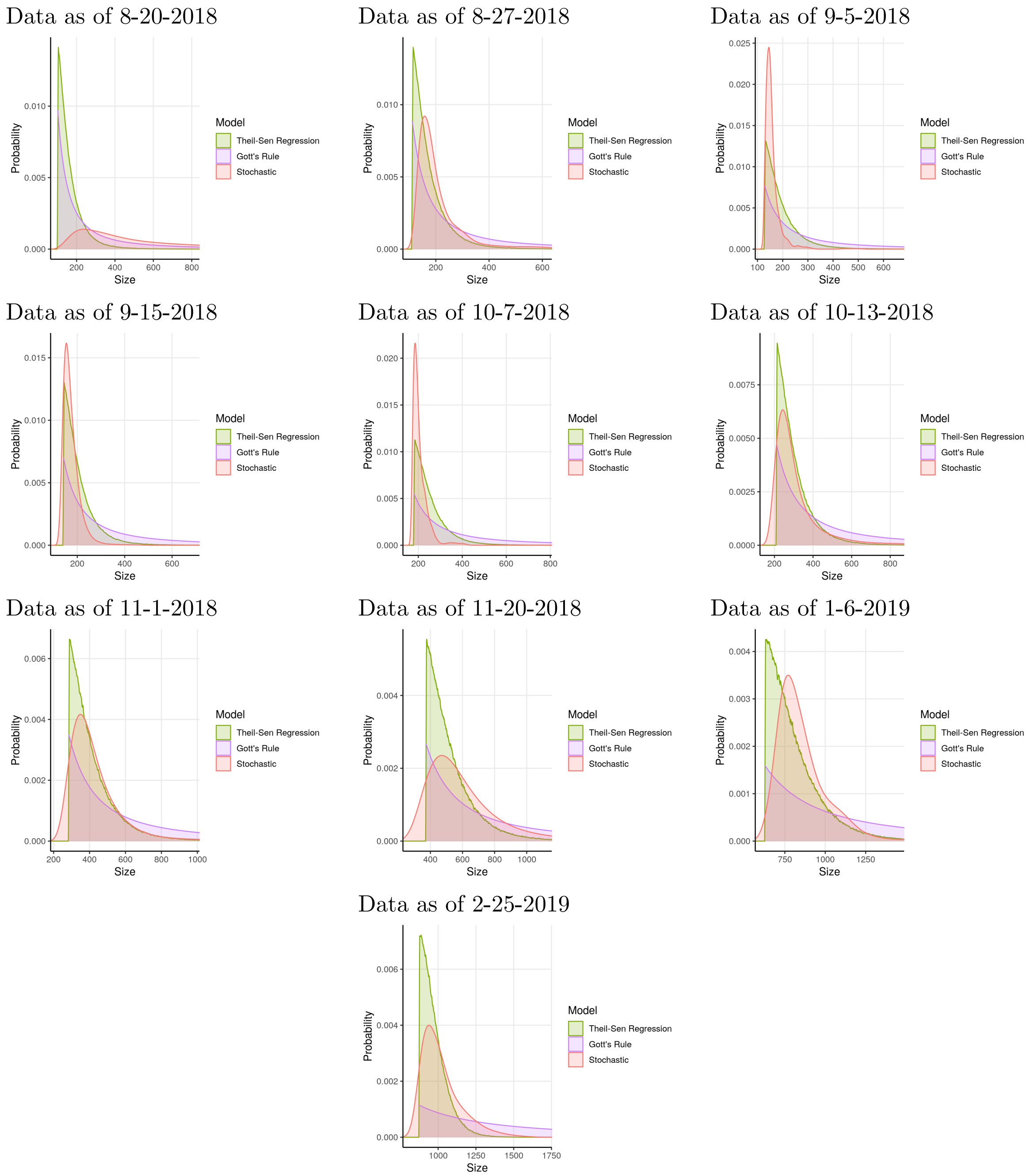
Data as of 8-20-2018
Data as of 8-27-2018
Data as of 9-5-2018
Data as of 9-15-2018
Data as of 10-7-2018
Data as of 10-13-2018
Data as of 11-1-2018
Data as of 11-20-2018
Data as of 1-6-2019
Data as of 2-25-2019
Probability
Size
Model
Theil-Sen Regression
Gott's Rule
Stochastic
